# How can vocal folds oscillate with a limited mucosal wave?

**DOI:** 10.1121/10.0014359

**Published:** 2022-10-03

**Authors:** Ingo R. Titze

**Affiliations:** Utah Center for Vocology, University of Utah, Salt Lake City, Utah 84112, USA ingo.titze@utah.edu

## Abstract

Self-sustained vocal fold vibration is possible with either or both of two mechanisms: (1) a mucosal wave propagating along the medial surface of the vocal folds and (2) a vocal tract that offers inertive reactance. A quantitative comparison shows the mucosal wave mechanism has a lower threshold pressure and a higher glottal efficiency, but the supraglottal inertance mechanism can assist in the oscillation and is effective in optimizing the two mechanisms. It is concluded that optimal parameters are a mucosal wave velocity on the order of 1 m/s and a diameter of the larynx canal (epilarynx tube) on the order of 0.8 cm.

## 1. Introduction

This express letter contains no new theories of vocal fold oscillation. Rather, it is intended for recognition and conceptualization of two independent mechanisms of self-sustained oscillation that are not often compared side-by-side. The simplest possible computational model is chosen for the phenomena of interest, one in which all the mathematical detail is provided with introductory-level aerodynamics and acoustics. All equations are ordinary differential equations. The model does not cover a broad range of frequency, intensity, or voice quality, nor is it intended for application to specific voice disorders. The primary purpose is to raise awareness about source-airway interaction. The paper should be useful to newcomers to the field, and it might be useful to clinicians who contemplate reconstructive surgery or behavioral intervention.

Two fundamentally different mechanisms of self-sustained oscillation of the vocal folds have been described mathematically for self-oscillating valves in airways (Titze, 1988; Fletcher, 1993). One is independent of the airway, while the other is critically dependent on the airway. Here, we will refer to them as the mucosal wave (MW) mechanism and the supraglottal inertance (SI) mechanism. They usually co-exist, but one or the other may be dominant. The MW mechanism requires an upward-moving MW on the medial surface of the vocal folds (Titze, 1988; Lucero *et al.*, 2011). The surface wave must propagate slowly enough so that inferior and superior portions of the vocal fold move out of phase (a half-wavelength mode pattern). This out of phase tissue movement produces alternating convergent-divergent glottal shapes, for which the glottal pressures offer an asymmetric force that is favorable for net energy transfer from the airstream to the vocal folds (Thomson *et al.*, 2005; Li *et al.*, 2006; Motie-Shirazi *et al.*, 2021). If this energy transfer is sufficient to overcome the viscous losses in vocal fold movement and deformation (including collision), self-sustained oscillation is achievable. However, if the mucosal tissue is not sufficiently pliable to support a slow-moving surface wave, the energy transfer diminishes, and self-sustained oscillation is more difficult to achieve. Most clinical approaches are focused on preserving or restoring the MW mechanism (e.g., Khosla *et al.*, 2008).

The SI mechanism, which can exist on its own or co-exist with the MW mechanism, involves the acoustic interaction of the airway with the vocal folds. It was first quantified with a computational model by Flanagan and Landgraf (1968). Synchronization is required between acoustic pressures in the vocal tract and surface pressures on the vocal folds. Much less is known about this mechanism in basic voice physiology than the MW mechanism, but it is well described in theories of oscillating valves and wind-instrument acoustics (Fletcher, 1993). It requires the airway of the vocal tract above the glottis to be acoustically inertive (air movement lagging in response to applied supraglottal pressure). In both mechanisms, a “strong push–weak push” alternates on the medial surface of the vocal folds to transfer energy from the airstream to the tissue.

For phonosurgery and voice therapy, a relevant question becomes whether or not airway modifications can be used to augment, or be a substitute for, un-achievable vocal fold tissue repair. The label “supraglottal hyperfunction” has been attached to compensatory behaviors that patients often exhibit when vocal fold tissues do not vibrate normally with the MW mechanism. Increasing vocal tract inertance requires narrowing a portion of the vocal tract, which may be interpreted as being similar to hyper-adduction of the vocal folds. Galindo *et al.* (2017) addressed the possibility of phonotrauma with source-filter interaction that included the larynx canal. Zhang (2021) showed that vocal fold contact pressures are affected by the epilaryngeal configuration. In a beneficial direction, Yanagisawa *et al.* (1989) showed that an aryepiglottic constriction contributed to a desirable ringing voice quality in operatic singing. Döllinger *et al.* (2006) and Döllinger *et al.* (2012) showed that vocal fold dynamics are influenced by the epilaryngeal (larynx canal) area. Kniesburges *et al.* (2017) have shown that the ventricular folds affect oscillation conditions. It will be shown here that optimizing the larynx canal diameter can facilitate self-sustained oscillation.

The limitations of this brief analysis are significant. A low-frequency analysis is chosen that contains no wave propagation and, therefore, no vowel effects. Concatenation of resistances and inertances of various airway sections is only an approximation. There is also no broad exploration with tissue morphology and lung pressure. These choices are deliberate, however, to focus on the nature of the self-oscillation phenomena with as few variables as possible.

## 2. Theoretical background

For vocal fold vibration, a MW has been described with a medial surface displacement *ξ* that varies in the vertical (*z*) direction as a traveling wave (Titze, 1988),

$$\xi \left(z,t\right)\;=\;\xi \;\left(t\;-\;z/v_{m}\right),$$ (1)

where *v_m_* is the MW velocity. It was then shown that by conducting a Taylor series expansion around a center point of the medial surface, the lower and upper margin displacements can be written as

$$\xi_{1}=\;\xi \;+\tau \;v_{g},$$ (2)

$$\xi_{2}=\;\xi \;-\tau \;v_{g},$$ (3)

where *ξ* is the center vocal fold tissue displacement, and *v_g_* is the center vocal fold tissue velocity *dξ/dt*. The variable *τ* is a delay time, defined in terms of the vocal fold thickness *T* and the MW velocity *v_m_* as

$$\tau =T/2v_{m}.$$ (4)

Two velocities, a lateral tissue velocity *v_g_* and a vertical wave velocity *v_m_*, define the upper and lower surface movement of the vocal folds. Hirano *et al.* (1981) have measured *v_m_* to be on the order of 1 m/s. It is largely determined by the elastic shear properties of the vocal fold mucosa. Without the vertical time delay *τ*, there can be no alternating convergent and divergent glottal shapes. The time delay produces strong driving pressures for glottal opening and weak driving pressures for glottal closing (Li *et al.*, 2006). Hence, *τ* is the key variable for the MW mechanism of self-sustained oscillation. No net energy can be imparted to the vocal folds by the glottal airflow with *τ* = 0 because the driving pressures in the opening and closing phases cancel each other.

The SI mechanism of self-sustained oscillation is based on the discovery that an inertive air column above the vocal folds produces a positive pressure if glottal flow is increasing and a negative pressure if glottal flow is decreasing, which is expressed mathematically as

P = I dU/dt, (5)

where *I* is a quantity known as inertance, and *U* is airflow into the vocal tract. Note that the rate of flow *dU/dt* is positive if flow is increasing (glottal opening) and negative if glottal flow is decreasing (glottal closing). This supraglottal pressure can transfer into the glottis and produce a “strong push–weak push” on the vocal folds for self-sustained oscillation.

## 3. Methods

**Fig. 1.**
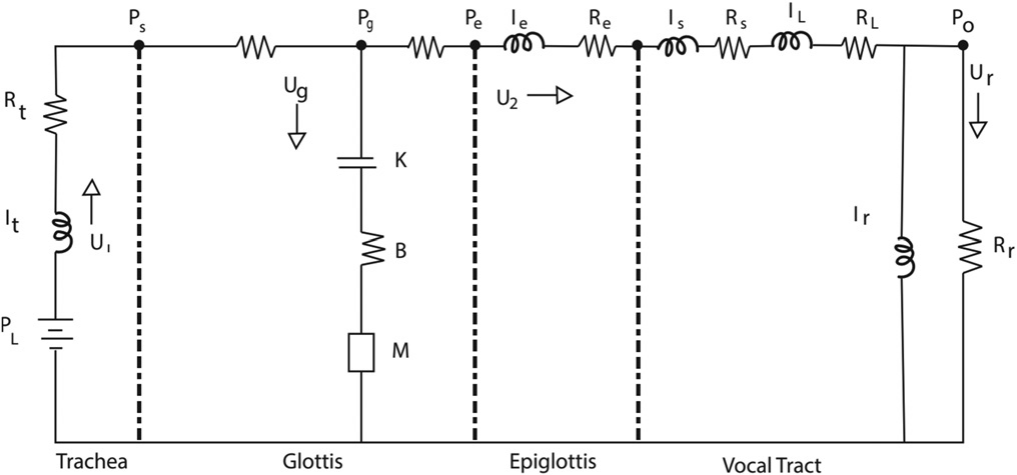
Low-frequency circuit diagram for quantifying self-sustained vocal fold oscillation.

Figure 1 shows an airflow and pressure diagram of the airway system for low frequencies, for which air compressibility is negligible. Wave propagation and acoustic vocal tract resonances are therefore neglected for frequencies on the order of 100–200 Hz. Incompressible airflow with acceleration and deceleration are included, however, because they have a major effect on vocal fold oscillation.

Consider *L* to be the length of the vocal folds, *T* the thickness, *M* the vocal fold mass, *K* the stiffness, *B* the damping coefficient (with a damping ratio 0.1), and *P_g_* the mean surface driving pressure. Pressures, resistances, inertances, and flows are as indicated in Fig. 1. The ordinary differential equations for the circuit are

$$P_{L}-\;R_{t}U_{1}-\;I_{t}dU_{1}/dt\;-\;P_{tg}-\;\left(R_{e}+R_{s}+R_{L}\right)U_{2}-\;\left(I_{e}+I_{s}+I_{L}\right)\;dU_{2}/dt\;–\;U_{r}R_{r}=\;0,$$ (6)

$$M\;d^{2}\xi /dt^{2}+\;B\;d\xi /dt\;+\;K\;\xi \;=\;P_{g}LT,$$ (7)

$$P_{o}=\;U_{r}R_{r}=\;I_{r}dU_{2}/dt\;–\;I_{r}dU_{r}/dt,$$ (8)

where *P_L_* is the lung pressure, *P_tg_* is the transglottal pressure, and *P_o_* is the oral pressure. The remaining quantities are resistances and inertances to be described below. Equation (7) is a second-order differential equation, which can be broken up into two first-order equations. Then the four first-order equations are solved with a fourth-order Runge–Kutta method. The independent variable is time, and the four dependent variables are vocal fold center displacement *ξ*, vocal fold center velocity *v_g_* = *dξ/dt*, glottal entry airflow *U_1_*, and radiation airflow *U_r_*. A continuity equation for glottal exit flows is

$$U_{2}=\;U_{1}–\;U_{g}.$$ (9)

The lumped-element resistances for low frequency can all be computed with a formula developed by Smith and Titze (2016) for tubes of varying lengths and diameters,

$$\begin{array}{c} R=\left(3.7631\times 10^{-7}\frac{Lt}{D^{4.4997}}+1.0268\times 10^{-6}\frac{1}{D^{4.0416}}\right)U+\left(3.9913\times 10^{-9}\frac{Lt}{D^{5.0089}}+8.0169\times 10^{-7}\frac{1}{D^{3.7696}}\right), \end{array}$$ (10)

where *Lt* is the tube length in m, *D* is the tube diameter in m, and *U* is the airflow in liters/s. The first term is the kinetic component, and the second is the viscous component. The density and viscosity of air are numerically included in the coefficients. The resistance *R* is expressed in Pa per liters/s, with a mean accuracy of ±6% according to the authors.

The lumped-element inertances for tubes have a simpler relation,

I = ρ L/A, (11)

where *ρ* is the density of air, *L* is the length, and *A* is the cross-sectional area of an equivalent circular airway section (Titze and Palaparthi, 2016). With Eq. (10), the tracheal resistance *R_t_*, the larynx canal resistance *R_e_* (the subscript denoting epi-larynx), the supraglottal tract resistance *R*_s_, and the lip resistance *R_L_* can be computed. With Eq. (11), the corresponding circuit inertances *I_t_*, *I_e_*, *I_s_*, and *I_L_* can be computed. The dimensions are provided in Table 1. The radiation resistance and inertance are, respectively,

$$R_{r}=128\varrho c/\left(9\pi^{2}A_{L}\right)$$ (12)

and

$$I_{r}=8\varrho /\left(3\pi^{2}D_{L}/2\right),$$ (13)

where *c* is the speed of sound, *D_L_* is the lip diameter, and *A_L_* is the lip area.

**Table 1.** Nominal parameter values in mathematical equations. Quantities in bold are the primary parameters for exploration and are listed with a range.

Airway diameters and lengths	Trachea: *D_t_* = 2 cm; *L_t_* = 15 cm	Larynx canal: ***D_e_* = 0.3–2 cm**; *L_e_* =2.5 cm	Supraglottal tract: *D_s_* = 3 cm; *L_s_* = 14 cm	Lips: *D_L_* = 1.0 cm; *L_L_* = 1.0 cm
Vocal fold parameters	Mass: *M* = 0.3 g	Stiffness: *K* = 3 N/cm	Damping ratio: ζ = 0.1	Medial surface: *L* = 1.0 cm; *T* = 0.6 cm
Other parameters	Adduction: *x*_01_ = 0.04 cm	Adduction: *x*_02_ = 0.04 cm	Mucosal wave: ***v_m_* = 0.4–12 m/s**	Lung pressure: *P_L_* = 1.0 kPa
Inertances in g/cm^4^ from Eq. (11)	Trachea: *I_t_* = 0.0054	Larynx canal at 0.8 cm diameter: *I_e_* = 0.0057	Supraglottal tract: *I_s_*= 0.0051; *I_L_* = 0.0015	Radiation *I_r_* = 0.000 62

As shown earlier in Eqs. (2)–(4), the mucosal wave on the medial surface of the vocal folds produces a time advancement *τ* at the lower margin of the vocal folds and an equivalent time delay *τ* at the upper margin, such that the entry, exit, and center glottal areas are

$$A_{g1}=\;2L\;\left(x_{01}+\;\xi_{1}\right),$$ (14)

$$A_{g2}=\;2L\;\left(x_{02}+\;\xi_{2}\right),$$ (15)

$$Ag\;=\;\left(A_{g1}+A_{g2}\right)/2,$$ (16)

where *x*_01_ and *x*_02_ are the lower (entry) and upper (exit) pre-oscillation positions.

The transglottal pressure is taken from an average glottal resistance of 4 kPa per liter/s measured on human subjects across gender, loudness, and adduction (Konnai *et al.*, 2017). Expressed in cgs units, which are used for convenience in the calculations, this resistance becomes 40 dyn/cm^2^ per cm^3^/s, so that

$$P_{tg}=\;40*U_{1}.$$ (17)

Subglottal and supraglottal pressures are

$$P_{s}=\;P_{L}-\;R_{t}U_{1}-\;I_{t}dU_{1}/dt$$ (18)

and

$$P_{e}=\;\left(R_{e}+R_{s}+R_{L}\right)\;U_{2}+\;\left(I_{e}+I_{s}+I_{L}\right)\;dU_{2}/dt\;+\;R_{r}U_{r}.$$ (19)

Finally, the vocal fold driving pressure is computed for five different glottal conditions. Beginning with the Bernoulli energy equation for pressures upstream of a flow detachment point, the pressure is

$$P\left(z\right)\;=\;P_{s}-\;P_{kd}a_{d}^{2}/a{\left(z\right)}^{2},$$ (20)

where *P_kd_* is the kinetic pressure (*1/2 ϱ v*^2^) at the detachment area *A_d_* (assumed to be at the center of the glottis for a divergent glottis and at glottal exit for a convergent glottis). *P_kd_* can further be expressed in terms of the transglottal pressure and a pressure recovery coefficient *k_e_* from detachment to glottal exit. In the equations to follow, *A*_min_ is the minimum allowed glottal area, *A_e_* is the larynx canal (epilarynx) area, *A_g_* is the area at the center of the glottis, and *P_g_* is the vocal fold driving pressure for Eq. (2):

For *A_g_*_1_ *> A_g_*_2_ and *A_g_*_2_ ≥ *A*_min_ (glottis open and convergent),

$$k_{e}=\;\text{max}\left[0,\;2\left(A_{g2}/A_{e}\right)\left(1-A_{g2}/A_{e}\right)\right];\;P_{kd}=\;\left(P_{s}-\;P_{e}\right)/\left(1-k_{e}\right);\;P_{g}=\;P_{s}-\;P_{kd}\left(A_{g2}/A_{g1}\right).$$ (21)

For *A_g1_* ≤ *A_g2_* and *A_g1_* ≥ *A*_min_ (glottis open and divergent),

$$k_{e}=\;\text{max}\left[0,\;2\left(A_{g}/A_{e}\right)\left(1-\;A_{g}/A_{e}\right)\right];\;P_{kd}=\;\left(P_{s}-\;P_{e}\right)/\left(1-\;k_{e}\right);\;P_{g}=\;P_{s}-\;P_{kd}\left(3A_{g1}+A_{g2}\right)/\left(4A_{g1}\right).$$ (22)

For *A_g_*_1_ *> A*_min_ and *A_g_*_2_ < *A*_min_ (glottis closed and convergent),

$$U_{2}=\;0;\;P_{g}=\;P_{s}.$$ (23)

For *A_g_*_1_ *< A*_min_ and *A_g_*_2_ > *A*_min_ (glottis closed and divergent),

$$U_{1}=\;0;\;U_{2}=\;-\;U_{g};\;P_{g}=\;P_{e}.$$ (24)

For *A_g_*_1_ *< A*_min_ and *A_g_*_2_ < *A*_min_ (glottis closed top and bottom),

$$U_{1}=\;0;\;U_{2}=\;0;\;P_{g}=\;\left(P_{s}+P_{e}\right)/2.$$ (25)

Table 1 shows the nominal values of the parameters in the equations above. The bolded values are the critical ones that were varied over a range.

The last row in Table I shows the inertances in g/cm^4^ calculated with Eq. (11). For the nominal 0.8 cm diameter and 2.5 cm length, the larynx canal (epilarynx tube) has the highest inertance. The trachea and the supraglottal tract have a slightly lower inertance due to their larger diameters, but greater lengths nearly equalize the inertances. The radiation inertance is an order of magnitude lower. The resistances are not tabulated because they are all airflow-dependent.

## 4. Results

Two parameters were varied over a wide range in the model, the larynx canal diameter *D_e_* and the MW velocity *v_m_*. These two distinguish the SI mechanism from the MW mechanism. The adduction parameters *x*_01_ and *x*_02_ were chosen to be 0.04 cm to approximate typical airflow rates for the glottal resistance and the larynx canal resistance (Titze, 2021; Zhang, 2021). In the current analytical model, the dynamically varying glottal resistance is also determined by glottal airflow [Eq. (17)].

### 4.1 SI mechanism

**Fig. 2.**
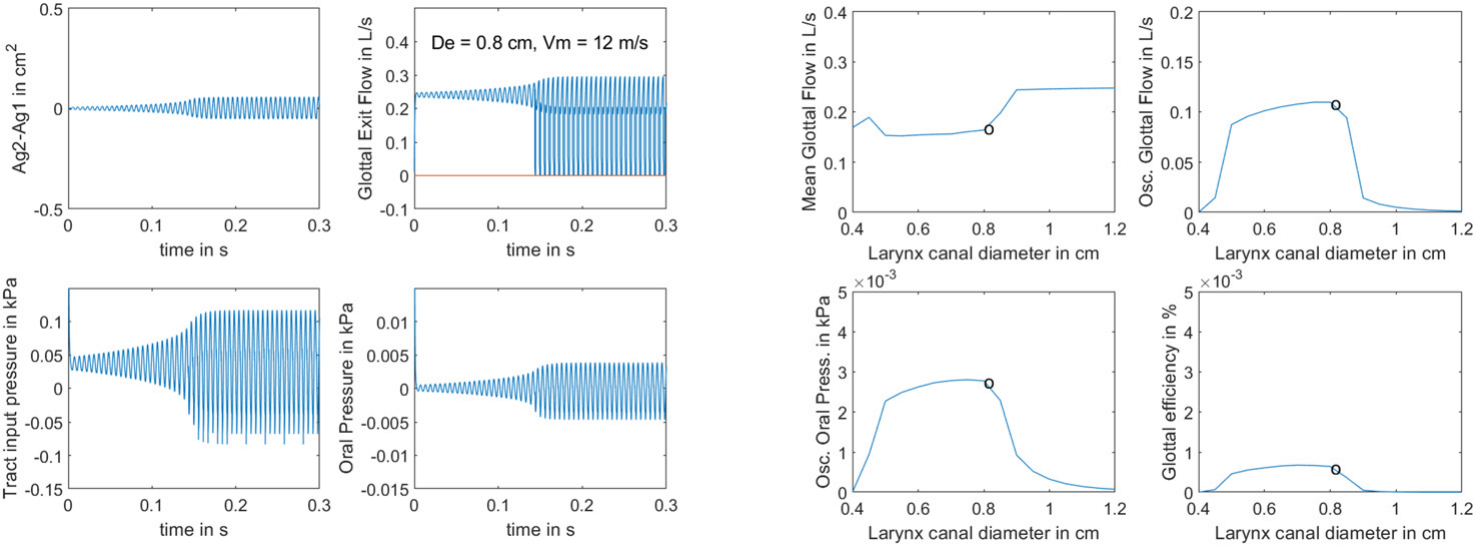
Left four panels, waveforms produced with *v_m_* = 12 m/s, *D_e_* = 0.8 cm, and *P_L_* = 1.0 kPa; right four panels, variation of outputs with a range of *D_e_.* Data points indicate where the waveforms on the left panels were selected.

To test the SI mechanism, 25 variations of larynx canal diameter *D_e_* were produced while the MW velocity was held at a high value of 12 m/s. This wave velocity produced minimal phase delay between upper and lower edge movement of the vocal folds, as seen in the upper left graph of the left panels in Fig. 2. The difference between the lower and upper glottal areas, *A_g_*_1_ − *A_g_*_2_, is plotted. This difference was never greater than 0.05 cm^2^ in either part of the glottal cycle (positive or negative). With a 0.6 cm vocal fold thickness, the convergence-divergence angle was less than 4.8°. According to Eq. (4), the time delay was 0.25 ms. With a fundamental frequency of vibration (*K/M*)^1/2^/2*π* = 159 Hz, the fundamental period was 6.29 ms, resulting in a phase delay of 0.25/6.29 of a period, or 14°. This is small compared to the 180° top-to-bottom (or 90° middle-to-top) phase delay often observed in vocal fold vibration. Note that the oscillation was slow to reach a steady state with vocal fold collision. It took about 150 ms. Voice onset time is a measure of “ease of phonation.” If it takes more than a few cycles, the lung pressure is only slightly above threshold. Pressures greater than 1.0 kPa did reduce the onset time, but they are not plotted here due to space limitations. In the remaining three graphs of the left four panels, it is seen that airflow peaked at about 0.3 liters/s, vocal tract input pressure oscillated between about −0.1 and +0.1 kPa, and oral pressure was about 20 times lower than vocal tract input pressure. This result is due to losses along the airway. The signals are all nearly sinusoidal because there is no wave propagation (and hence no vocal tract resonance) for this low-frequency analysis with incompressible airflow.

The right four panels of Fig. 2 show *post hoc* calculations for a group of 25 simulations in which the larynx canal diameter *D_e_* was varied from 0.4 to 1.2 cm, a range that bracketed cases where glottal closure occurred (*D_e_* = 0.5–0.8 cm). The MW velocity was kept constant at the high value of 12 m/s. Very small larynx canal diameters (*D_e_* ≤ 0.3 cm) did not produce self-sustained oscillation with the 1.0 kPa lung pressure. On the other extreme, diameters larger than 0.8 cm also required larger than 1.0 kPa lung pressure. Data circles on the graphs indicate where the waveforms on the left panels were selected. As seen in the figure, mean glottal airflow was lowest in the region where collision occurred, while oscillating glottal airflow, oscillating oral pressure, and glottal efficiency all increased slightly with *D_e_*. Glottal efficiency reached a peak at 0.8 cm and then declined with declining oscillating pressures. This efficiency was calculated as the ratio of RMS output power $P_{o}^{2}$*/R_r_* divided by the aerodynamic power *P_L_U_1_* available from the lungs. An important result is that glottal efficiency can be optimized with a mid-range larynx canal (epilarynx) diameter. For smaller diameters, the airway resistances (and corresponding energy losses) are too high, while for large diameters, the mean airflow is too high and the inertances are too low.

### 4.2 MW mechanism

**Fig. 3.**
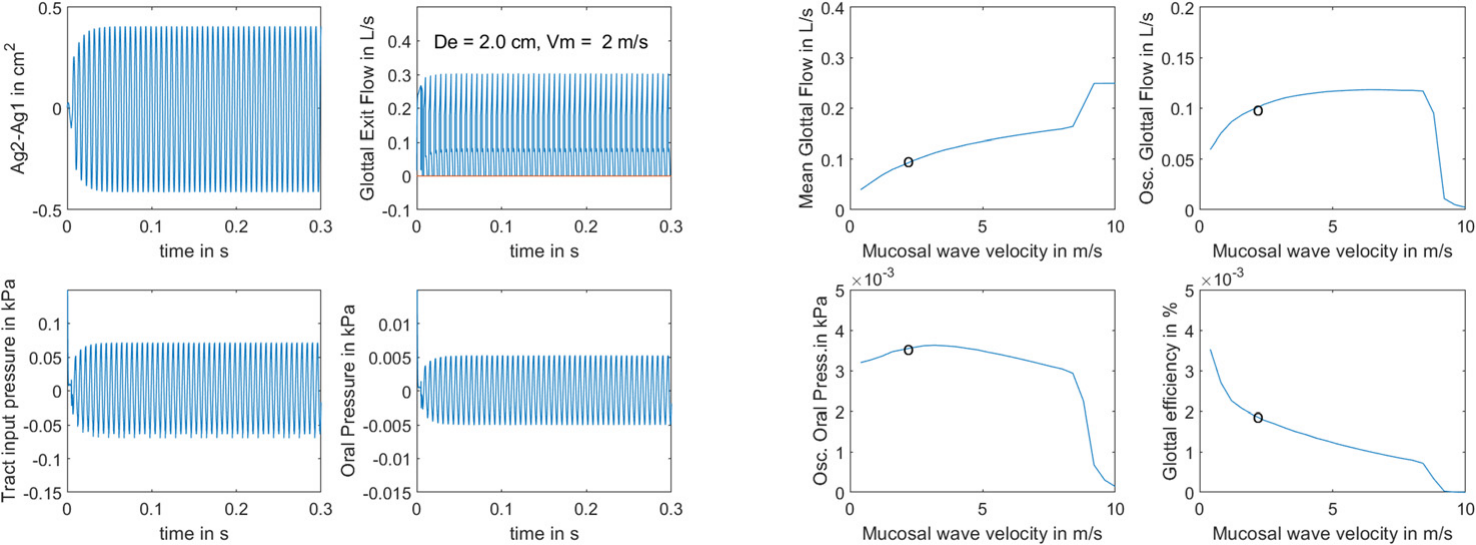
Left four panels, waveforms produced with *v_m_* = 2 m/s, *D_e_* = 2.0 cm, and *P_L_* = 1.0 kPa; right four panels, output variations with a range of *v_m_*. Data points indicate where the waveforms on the left panels were selected.

The second experiment involved *v_m_* as the parameter, the MW velocity. This parameter was more effective in producing self-sustained oscillation because the phonation threshold pressure was lower. As Fig. 3 (left four panels) shows, onset time to vocal fold collision was only about 20 ms, or about 5 cycles. The larynx canal diameter was held steady at a large value of 2.0 cm, while the MW velocity was a mid-range value of 2.0 m/s. The scale of the waveforms was kept the same as in Fig. 2. The most striking difference is the (*A_g_*_1_ − *A_g_*_2_) waveform in the top left graph of the left panels. This indicates that alternating convergent-divergent shapes are present in equal amounts. With a 2 m/s MW velocity, the time delay between top and bottom was 0.75 ms, the phase delay was 42°, and the convergence-divergence angle was 14.4°. By reducing the wave velocity to 0.5 m/s, the phase delay becomes 168°, the convergence-divergence angle becomes 57.6°, and the (*A_g_*_1_ − *A_g_*_2_) waveform amplitude grows by a factor of 4, off the scale in Fig. 3.

The right four panels of Fig. 3 show *post hoc* calculations on 25 simulations with the MW velocity *v_m_* as the variable. Data circles show where the waveforms on the left panels were selected. Note the general decline of glottal efficiency with increasing MW velocity. The decline is steepest in the 0.5–2.0 m/s range. However, a broad range of MW velocities can produce self-sustained oscillation. No oscillation was achieved with values greater than 8 m/s, but different parameter sets (increased or decreased adduction, mass, stiffness, lung pressure) will likely produce different oscillation regions. In contrast to the SI mechanism, there is no mid-range optimum wave velocity. According to Eq. (4), the minimum *v_m_* for a half-period (180°) bottom-top delay can be related to the vocal fold thickness *T* and the fundamental frequency *f_o_*,

$$v_{m}=\;\left(T/2\right)\;(1/\tau )\;=\;\left(T/2\right)\;\left(2f_{o}\right)\;=\;f_{o}T.$$ (26)

Any value less than *f_o_T* would produce a phase delay greater than 180° between top and bottom of the vocal folds, which would diminish the alternating convergent-divergent glottal shapes. For the chosen values *f_o_* = 159 Hz and *T *=* *0.6 cm, this minimum *v_m_* value should be 0.954 m/s. Note that this is the lowest value on the right panel graphs of Fig. 3. As *f_o_* is raised in phonation, *T* usually decreases, which means that *v_m_* and vertical phase can remain relatively constant, at least over a small *f_o_* range. However, if *f_o_* doubles or triples, it is unlikely that *T* can decrease proportionately.

## 5. Discussion and conclusions

Both the MW mechanism and the SI mechanism allow an alternating “strong push–weak push” on the vocal fold surfaces for self-sustained oscillation. The two usually co-exist in normal voice production, as was shown here, but the MW mechanism is dominant for normal tissue conditions. Nature has provided a soft, gel-like mucosa for surface wave motion that allows alternating convergent–divergent glottal shapes for low phonation pressure and “ease of phonation.” A MW velocity in the range of 0.5–4 m/s provides appropriate convergence-divergence angles, with the value *f_o_T* (fundamental frequency in Hz times vocal fold thickness in m) being the ideal wave velocity. Higher wave velocities limit the effectiveness of the MW mechanism.

Nature has also provided a narrow larynx canal for favorable supraglottal source-airway interaction. A larynx canal diameter around the value 0.8 cm appears optimal. The equivalent cross-sectional circular area is 0.5 cm^2^, a typical value reported by Story *et al.* (1996) on normal subjects. It is not entirely clear to what degree the laryngo-pharyngeal musculature can actively alter the diameter without compromising breathing and swallowing, but the epiglottis can move in the anterior-posterior direction, and its geometry can perhaps be rounded into an omega-shape. The false folds can also be adducted, but that may produce undesirable false-fold oscillation. Much more work is needed to optimize the overall airway configuration. The current simplified model may pave the way for more sophisticated finite-element modeling with large sets of parameter variations.
